# Functional Characterization of the 1,5-Benzodiazepine Clobazam and Its Major Active Metabolite *N*-Desmethylclobazam at Human GABA_A_ Receptors Expressed in *Xenopus laevis* Oocytes

**DOI:** 10.1371/journal.pone.0120239

**Published:** 2015-03-23

**Authors:** Harriet Hammer, Bjarke Ebert, Henrik Sindal Jensen, Anders A. Jensen

**Affiliations:** 1 Department of Drug Design and Pharmacology, Faculty of Health and Medical Sciences, University of Copenhagen, Copenhagen, Denmark; 2 H. Lundbeck A/S, Valby, Denmark; McLean Hospital/ Harvard Medical School, UNITED STATES

## Abstract

The 1,5-benzodiazepine clobazam is indicated for the adjunctive treatment of seizures associated with Lennox-Gastaut syndrome in patients 2 years of age or older in the United States, and for treatment of anxiety and various forms of epilepsy elsewhere. Clobazam has been reported to exhibit different *in vivo* adverse effects and addiction liability profile than the classic 1,4-benzodiazepines. In this study, it was investigated whether the *in vitro* pharmacological properties of clobazam and its major active metabolite *N*-desmethylclobazam could explain some of these clinical differences. The functional properties of the two 1,5-benzodiazepines were characterized at the human γ-aminobutyric acid type A receptor (GABA_A_R) subtypes α_1_β_2_γ_2S_, α_2_β_2_γ_2S_, α_3_β_2_γ_2S_, α_5_β_2_γ_2S_ and α_6_β_2_δ expressed in *Xenopus laevis* oocytes by use of two-electrode voltage-clamp electrophysiology and compared to those exhibited by the 1,4-benzodiazepine clonazepam. All three compounds potentiated GABA EC_20_-evoked responses through the α_1,2,3,5_β_2_γ_2S_ GABA_A_Rs in a reversible and concentration-dependent manner, with each displaying similar EC_50_ values at the four subtypes. Furthermore, the degrees of potentiation of the GABA EC_20_ currents through the four receptors mediated by saturating modulator concentrations did not differ substantially for any of the three benzodiazepines. The three compounds were substantially less potent (200-3900 fold) as positive allosteric modulators at the α_6_β_2_δ GABA_A_R than at the α_1,2,3,5_β_2_γ_2S_ receptors. Interestingly, however, clobazam and especially *N*-desmethylclobazam were highly efficacious potentiators of α_6_β_2_δ receptor signaling. Although this activity component is unlikely to contribute to the *in vivo* effects of clobazam/*N*-desmethylclobazam, the 1,5-benzodiazepine could constitute an interesting lead for novel modulators targeting this low-affinity binding site in GABA_A_Rs. In conclusion, the non-selective modulation exerted by clobazam, *N*-desmethylclobazam and clonazepam at the α_1_β_2_γ_2S_, α_2_β_2_γ_2S_, α_3_β_2_γ_2S_ and α_5_β_2_γ_2S_ GABA_A_Rs indicate that the observed clinical differences between clobazam and 1,4-benzodiazepines are likely to arise from factors other than their respective pharmacological properties at the GABA_A_Rs as investigated here.

## Introduction

As the main inhibitory neurotransmitter in the central nervous system (CNS), γ-aminobutyric acid (GABA) is directly involved in, or contributes to, an exhaustive number of physiological processes and pathophysiological states. GABA exerts its effects through two receptor classes, the GABA_A_ and GABA_B_ receptors [1, 2]. The GABA_A_ receptors (GABA_A_Rs) are membrane-bound, chloride-permeable ligand-gated ion channels belonging to the Cys-loop receptor superfamily, which also includes receptors for acetylcholine, serotonin, and glycine [2–5]. The GABA_A_R complex is composed of five subunits, and the existence of a total of 19 human GABA_A_ subunits (α _1–6_, β_1–3_, γ _1–3_, δ, ε, π, θ and ρ_1–3_) gives rise to an array of physiologically relevant receptor subtypes [6]. It is estimated that approximately 80% of all GABA_A_Rs are α βγ receptors predominantly composed of α _1/2/3/5_, β_2/3_, and γ _2_ subunits in an anticlockwise α β α γ β arrangement (viewed from the extracellular space) [6–9]. However, numerous other physiologically important receptor subtypes exist, including the α _4_βδ and α _6_βδ receptors that through their predominant expression as extra- and perisynaptic receptors are key mediators of the GABAergic tonic inhibition [10, 11].

The signaling through the GABA_A_R is susceptible to modulation by numerous ligands acting through different allosteric sites in the receptor complex, and many of these ligands are used to treat human pathologies [12–16]. Delineation of the molecular modes of action of these modulators at the receptors has provided considerable insight into the signal transduction mechanism of the GABA_A_R as well as the molecular compositions of its allosteric sites. The allosteric modulation of the α _1,2,3,5_βγ GABA_A_Rs exerted by benzodiazepines is predominantly mediated through a high-affinity binding site located in the extracellular α ^(+)^/γ ^(–)^ subunit interface of the receptor [14, 17]. However, benzodiazepines have also been proposed to target a low-affinity binding site located in the transmembrane domains of both αβ and αβγ GABA_A_Rs [18]. Thus, the benzodiazepines have been proposed to possess a nM activity component arising exclusively from α _1,2,3,5_βγ GABA_A_Rs and a μM activity component that potentially could involve all GABA_A_Rs [18]. Furthermore, several recent studies have proposed the existence of a low-affinity binding site for some benzodiazepines and other benzodiazepine-site ligands such as CGS 9895, LAU 177 and Ro 15–4513 in the extracellular α ^(+)^/β^(–)^ subunit interface of the GABA_A_R, a site homologous to the extracellular high-affinity α^(+)^/γ^(–)^ binding site for benzodiazepines in the αβγ GABA_A_R [19–23].

The selectivity profile of a particular benzodiazepine at the different α_1,2,3,5_βγ GABA_A_R subtypes is believed to correlate to its clinical efficacy and adverse effects. Insights gained from studies of knock-in mice expressing benzodiazepine-insensitive subtypes have provided the rationale for the development of positive allosteric modulators (PAMs) of α_1_-containing subtypes as hypnotics, of PAMs of α _2_- or α_3_-containing subtypes as anxiolytics and analgesics, and of negative allosteric modulators (NAMs) of α _5_-containing receptors as cognitive enhancers [12, 13, 15, 16, 24]. Moreover, other studies suggest that the anticonvulsive and anxiolytic effects of benzodiazepines may be mediated via α _2_-containing GABA_A_Rs, whereas modulation of these subtypes has little or no sedative effects [25–29]. However, experiments in rodents indicate that the anticonvulsant effects require modulation of more than one specific α-subunit-containing GABA_A_R and that modulation of different subtypes may act synergistically [29].

**Fig 1:**
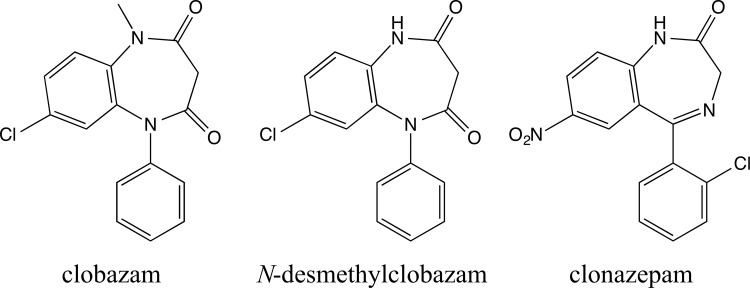
Chemical structures of clobazam, *N*-desmethylclobazam and clonazepam.

To date, most benzodiazepines reported capable of modulating GABA_A_R signaling are 1,4-benzodiazepines. One of the few exceptions is the 1,5-benzodiazepine clobazam (Fig. 1). Clobazam (Onfi) has recently been approved in the United States and is indicated for the adjunctive treatment of seizures associated with Lennox-Gastaut syndrome in patients aged 2 years and older, and outside the United States the drug is routinely administered for anxiety disorders and epilepsy [30–32]. Interestingly, clobazam has been reported to exhibit different *in vivo* adverse effects and addiction liability profile than the 1,4-benzodiazepines, including clonazepam (Klonopin, Fig. 1), an antiepileptic drug approved for the treatment of Lennox-Gastaut syndrome (petit mal variant), and akinetic and myoclonic seizures. For example, studies suggest that clobazam induces fewer psychomotor disturbances than clonazepam when dosed at clinically effective concentrations in healthy volunteers [33, 34]. Moreover, preclinical studies suggest that clobazam in contrast to clonazepam and other 1,4-benzodiazepines may exert more specific anticonvulsant/antiepileptic over sedative effects [35, 36]. The major active metabolite of clobazam, *N*-desmethylclobazam (Fig. 1), has been shown to have a longer plasma half-life than the parent compound (79 h vs. 36 h), resulting in greater metabolite plasma concentrations following long-term clobazam dosing in humans [37]. Hence, the metabolite is likely to contribute significantly to the clinical efficacy of clobazam, and interestingly *N*-desmethylclobazam has been reported to produce fewer adverse effects than clobazam [38, 39].

Considering that clobazam has been administered for various indications in the clinic for decades, the current insight into the pharmacological characteristics of clobazam and *N*-desmethylclobazam at GABA_A_Rs is surprisingly limited [40]. In a recent study, we delineated the binding characteristics of clobazam, *N*-desmethylclobam and the 1,4-benzodiazepine clonazepam at native GABA_A_Rs in rat brain membranes and at human α _1,2,3,5_β_2_γ_2S_GABA_A_Rs expressed in HEK293 cells in a [^3^H]flumazenil competition binding assay [41]. In the present study, we have characterized the functional properties of clobazam and *N*-desmethylclobazam at human α _1_β_2_γ_2S_, α _2_β_2_γ_2S_, α _3_β_2_γ_2S_, α _5_β_2_γ_2S_ and α _6_β_2_δ GABA_A_Rs expressed in *Xenopus laevis* oocytes using two-electrode voltage clamp (TEVC) electrophysiology and compared these functionalities to that exhibited by clonazepam.

## Materials and Methods

### Materials

GABA, ZnCl_2_ and chemicals for buffers were obtained from Sigma-Aldrich (Denmark), and DS2 was obtained from Tocris Cookson (Bristol, UK). Clobazam (synthesized at H. Lundbeck A/S, Denmark), *N*-desmethylclobazam (from Johnson Matthey Pharma Services, MA, USA), clonazepam (from Lipomed AG, Switzerland), diazepam, and zolpidem (both from Sigma-Aldrich, Denmark) were dissolved in DMSO and diluted in Ringer buffer on the given experimental day. The cDNAs encoding human α_1_, α_2_, α_3_, α_5_, α_6_, β_2_, γ_2S_ and δ GABA_A_R subunits were kind gifts from Dr. P J Whiting and Merck, Sharp & Dohme (Harlow, Essex, UK), and they were subcloned into mammalian expression vector pcDNA3.1 (Invitrogen, Denmark) as described previously [42, 43].

### Preparation of cRNA and injection in *Xenopus laevis* oocytes

The cDNAs encoding the human GABA_A_R subunits were linearized with *Dra*III (α_1_, α_2_, α_3_, α_5_ and α_6_), *Sma*I (β_2_ and γ_2S_) or *Stu*I (δ) and used as templates for *in vitro* cRNA synthesis using the T7 mMESSAGE mMACHINE High Yield Capped RNA Transcription Kit (Life Technologies Corporation, Carlsbad, CA, USA). Except where otherwise indicated, *Xenopus* oocytes were injected with 36.8 nL cRNA solution encoding for α_1_β_2_γ_2S_, α_2_β_2_γ_2S_, α_3_β_2_γ_2S_ and α_5_β_2_γ_2S_ GABA_A_Rs in a 1:1:1 α:β_2_:γ_2S_ ratio (2.7 ng/μL of each subunit), with 46 nL cRNA solution encoding for the α_6_β_2_δ GABA_A_R in a 10:1:10 α_6_:β_2_:δ ratio (1:0.1:1 μg/μL), with 18 nL cRNA solution encoding for the α_1_β_2_ GABA_A_R in a 1:1 α_1_:β_2_ ratio (0.6:0.6 μg/μL), or with 46 nL cRNA encoding for α_6_β_2_ (α_6_:β_2_ ratio: 1:0.1 μg/μL). Following injection, the oocytes were incubated at 18°C in modified Barth’s solution [88 mM NaCl, 1 mM KCl, 15 mM HEPES (pH 7.5), 2.4 mM NaHCO_3,_ 0.41 mM CaCl_2_, 0.82 mM MgSO_4_, 0.3 mM Ca(NO_3_)_2_, 100 U/mL penicillin and 100 μg/mL streptomycin]. Electrophysiological recordings were performed 3 to 6 days after injection.

### Electrophysiological recordings

Electrophysiological recordings were performed using the TEVC technique on *Xenopus* oocytes expressing various GABA_A_R combinations using a protocol adapted from previous studies [44, 45]. Oocytes were placed in a recording chamber and gravity perfused with Ringer buffer [115 mM NaCl, 2.5 mM KCl, 10 mM HEPES (pH 7.5), 1.8 mM CaCl_2_, 0.1 mM MgCl_2_]. Cells were impaled with agar-plugged 0.5–1 MΩ electrodes containing 3 M KCl and voltage clamped at −70 mV by a Gene Clamp 500B amplifier (Axon Instruments, Union City, CA, USA) and recorded with pClamp 10 (Windows version, Molecular Devices, LLC, Sunnyvale, CA, USA). The oocytes were continuously perfused with Ringer buffer, and the test compounds were applied in the perfusate. Experiments were performed at room temperature and each data point represents the mean ± S.E.M. value of recordings performed on at least two oocytes from at least two different batches of oocytes. The recorded baseline-to-peak current amplitudes were analyzed using Clampfit 10.1 (Axon Instruments, Union City, CA, USA). Analogously to the procedures used in a recent study [43], the incorporation of the γ_2S_ subunit into the GABA_A_Rs assembled at the cell surface of α_1,2,3,5_β_2_γ_2S_-expressing oocytes was confirmed on a routinely basis with 100 μM ZnCl_2_ [46]. The presence of δ in cell surface-expressed receptors in α_6_β_2_δ-injected oocytes was confirmed on a routinely basis using the δ-GABA_A_R selective PAM DS2 (1 μM) [47]. Furthermore, taking advantage of the differential sensitivity of αβ and αβδ receptors to Zn^2+^ as antagonist [45, 48–50], 1 μM ZnCl_2_ was used to verify that a homogenous population of ternary α_6_β_2_δ receptor complexes was expressed in these oocytes.

The GABA EC_20_ (the GABA concentration eliciting 20% of the maximum effect) for each receptor subtype was determined on two oocytes on each day of the experiment. A maximum concentration of GABA was applied until the peak of the response was observed, usually within 30 seconds. When two consecutive applications of the maximum GABA concentration were observed to elicit responses of similar effect (± 5%), 3–4 different concentrations of GABA were applied to the perfusate until the peak of the response was observed. Two to five minutes of wash time between each application were allowed to prevent receptor desensitization. Data for the GABA concentrations was normalized to the maximal response elicited by GABA on each oocyte, and the concentration-response curves were fitted in Prism GraphPad 5.0a (GraphPad Software, Inc. La Jolla, CA, USA) by nonlinear regression using the equation for sigmoidal dosage-response with variable slope (Equation 1): (1) Y = Bottom + [(Top—Bottom)/(1 + 10^(logEC50-X)Hillslope^)].

Bottom = response at the bottom plateau; EC_50_ = concentration giving rise to 50% of the maximum response; Top = response at the top plateau; X = logarithm of the concentration; Y = response.

The GABA EC_20_ response was calculated using Equation 2 (F = 20), and this concentration was subsequently used on a given experimental day. (2) log EC_50_ = (1/HillSlope) [log (F/(100-F)].

The functional characteristics of clobazam, *N*-desmethylclobazam, clonazepam, diazepam, and zolpidem on the GABA_A_Rs were determined by co-application of different concentrations of the compounds with GABA EC_20_. The test compounds were pre-applied 30 seconds prior to the co-application with GABA EC_20_. At the end of an experiment, a maximum concentration of GABA was applied in the perfusate to determine the maximum response elicited by GABA through the receptor, which served as the internal standard and as a control of any potential drift in the system during the recordings. Two to five minutes of wash time between each application were permitted to overcome receptor desensitization. Data for the benzodiazepines was normalized to the responses elicited by GABA EC_20_ at the receptor (the EC_20_ response was defined as 100%). Concentration-response curves for the benzodiazepines were fitted using Equation 1.

### Data analysis

Using GraphPad Prism 4 (GraphPad Software, Inc., La Jolla, CA, USA), the pEC_50_ and I_max_ values were evaluated for statistical differences across the receptor subtypes per compound using one-way ANOVA with Tukey’s Multiple-Comparison *Post-hoc* Test, where *P*<0.05 was considered significant.

## Results

**Fig 2:**
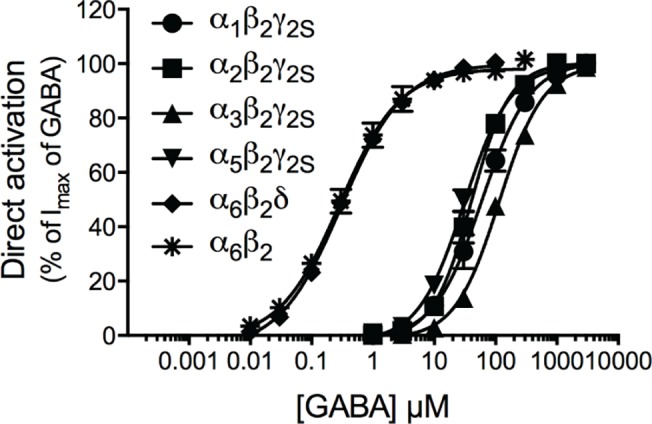
Functional properties of GABA at six human GABA_A_Rs expressed in *Xenopus* oocytes. Concentration-response curves of GABA at the α_1_β_2_γ_2S_ (circle), α_2_β_2_γ_2S_ (square), α_3_β_2_γ_2S_ (triangle), α_5_β_2_γ_2S_ (inverted triangle), α_6_β_2_δ (diamond) and α_6_β_2_ (asterisk) GABA_A_Rs (means ± S.E.M.; N = 4–7).

Functional characterization of GABA and determination of GABA EC_20_ values at human α_1_β_2_γ_2S_, α_2_β_2_γ_2S_, α_3_β_2_γ_2S_, α_5_β_2_γ_2S_ and α_6_β_2_δ GABA_A_Rs expressed in *Xenopus* oocytes. Prior to the functional characterization of the benzodiazepines at the five GABA_A_R subtypes, the pharmacological properties of GABA at the receptors were determined. The concentration-response relationships displayed by GABA at the receptors are given in **Fig.** 2, and the pharmacological data are summarized in Table 1. The EC_50_ and Hill slope values determined for the agonist were in good agreement with those observed in previous studies of these five receptors expressed in *Xenopus* oocytes [46, 51].

**Table 1:** Functional properties of GABA at the human α_1_β_2_γ_2S_, α_2_β_2_γ_2S_, α_3_β_2_γ_2S_, α_5_β_2_γ_2S_, α_6_β_2_δ and α_6_β_2_GABA_A_Rs expressed in *Xenopus* oocytes. The EC_50_ values are given in μM with pEC_50_ ± S.E.M. values in brackets, and the Hill slopes (n_H_ ± S.E.M.) and the numbers of experiments (N) are also given.

Receptor	EC_50_ [pEC_50_ ± S.E.M.]	n_H_ ± S.E.M.	N
α_1_β_2_γ_2S_	57 [4.25 ± 0.10]	1.19 ± 0.05	6
α_2_β_2_γ_2S_	40 [4.40 ± 0.07]	1.44 ± 0.07	4
α_3_β_2_γ_2S_	120 [3.94 ± 0.03]	1.22 ± 0.05	7
α_5_β_2_γ_2S_	31 [4.50 ± 0.06]	1.19 ± 0.08	6
α_6_β_2_δ	0.30 [6.52 ± 0.04]	0.89 ± 0.02	6
α_6_β_2_	0.29 [6.54 ± 0.07]	0.89 ± 0.09	7

For the functional characterization of the benzodiazepines at the receptors, the GABA EC_20_ values were determined on the days of the experiments. The actual GABA concentrations constituting the EC_20_ values for the respective receptor subtypes varied within 2- to 4-fold from day to day (α_1_β_2_γ_2S_: 20–30 μM; α_2_β_2_γ_2S_: 25–45 μM; α_3_β_2_γ_2S_: 25–60 μM; α_5_β_2_γ_2S_: 15–45 μM; α_6_β_2_δ0.05–0.20 μM). Thus, this procedure enabled us to use very accurate GABA EC_20_ concentrations for these studies. In fact, retrospective evaluation of the specific GABA concentrations used for characterization of the benzodiazepines in these subsequent studies revealed that these varied very little from the calculated EC_20_ (as percentage of GABA I_max_, means ± S.E.M., N): α_1_β_2_γ_2S_ (20.2 ± 0.74, N = 15); α_2_β_2_γ_2S_ (19.8 ± 0.89, N = 17); α_3_β_2_γ_2S_ (21.0 ± 1.07, N = 11); α_5_β_2_γ_2S_ (18.6 ± 1.30, N = 13); and α_6_β_2_δ (15.7 ± 1.47, N = 16).

**Fig 3:**
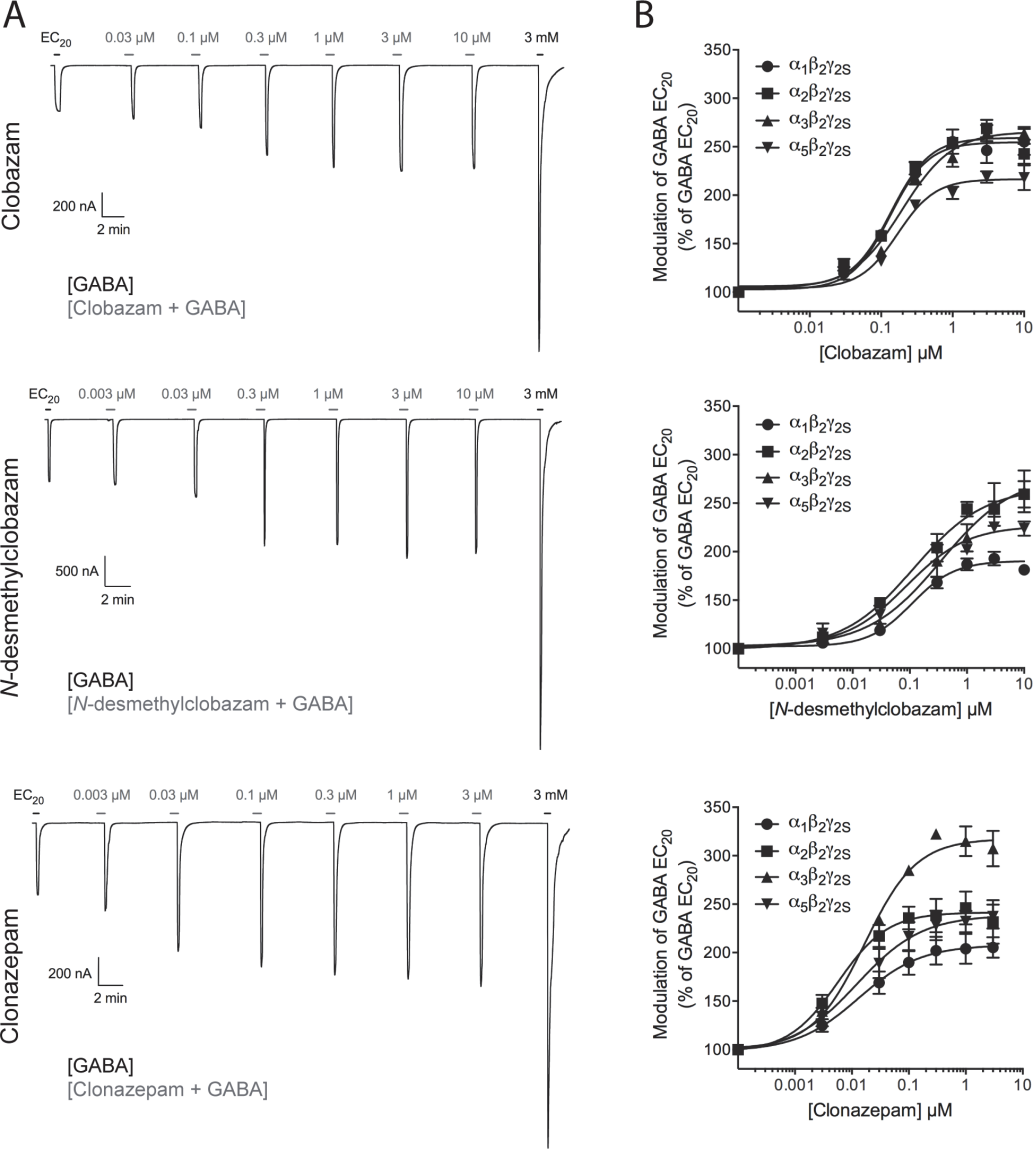
Functional properties of clobazam, *N*-desmethylclobazam and clonazepam at four human GABA_A_Rs expressed in *Xenopus* oocytes. (A) Representative traces for various concentrations of clobazam (top), *N*-desmethylclobazam (middle) and clonazepam (bottom) co-applied with GABA EC_20_ to oocytes expressing the α_5_β_2_γ_2S_ GABA_A_R. The black bars represent applications of GABA EC_20_ and of 3 mM GABA that elicits maximal current through the receptor. The grey bars represent applications of various concentrations of clobazam, *N*-desmethylclobazam or clonazepam (a 30 s pre-incubation with the compound followed by co-application of the compound and GABA EC_20_). (B) Concentration-response relationships for clobazam (top), *N*-desmethylclobazam (middle) and clonazepam (bottom) at α_1_β_2_γ_2S_, α_2_β_2_γ_2S_, α_3_β_2_γ_2S_ and α_5_β_2_γ_2S_ GABA_A_Rs in the presence of GABA EC_20_ (means ± S.E.M.; N = 2–6).

Functional properties of clobazam, *N*-desmethylclobazam, and clonazepam at human α_1_β_2_γ_2S_, α_2_β_2_γ_2S_, α_3_β_2_γ_2S_, α_5_β_2_γ_2S_ and α_6_β_2_δ GABA_A_Rs expressed in *Xenopus* oocytes. The functional properties of clobazam, *N*-desmethylclobazam, and clonazepam when preincubated and co-applied with EC_20_ GABA at the four α_1,2,3,5_β_2_γ_2S_ GABA_A_Rs expressed in *Xenopus* oocytes are given in Table 2. Representative traces for each of the three compounds at the α_5_β_2_γ_2S_subtype are given in Fig. 3A, and the concentration-response relationships obtained for the three compounds at the four receptors are outlined in Fig. 3B.

**Table 2:** Functional properties of clobazam, *N*-desmethylclobazam, and clonazepam at the human α_1_β_2_γ_2S_, α_2_β_2_γ_2S_, α_3_β_2_γ_2S_, α_5_β_2_γ_2S_, α_6_β_2_δ and α_6_β_2_GABA_A_Rs expressed in *Xenopus* oocytes.

Receptor	EC_50_ [pEC_50_ ± S.E.M.]	I_max_ ± S.E.M. (%)	N
Clobazam			
α_1_β_2_γ_2S_	132 [6.88 ± 0.05]	256 ± 12	5
α_2_β_2_γ_2S_	138 [6.86 ± 0.04]	261 ± 6.7	6
α_3_β_2_γ_2S_	240 [6.62 ± 0.10]	269 ± 0.8	4
α_5_β_2_γ_2S_	174 [6.76 ± 0.03]	216 ± 7.0	6
α_6_β_2_δ	55,000 [4.26 ± 0.04]	528 ± 46	6
*N*-desmethylclobazam			
α_1_β_2_γ_2S_	151 [6.82 ± 0.07]	203 ± 8.5	6
α_2_β_2_γ_2S_	138 [6.86 ± 0.18]	270 ± 20	6
α_3_β_2_γ_2S_	282 [6.55 ± 0.11]	270 ± 27	5
α_5_β_2_γ_2S_	98 (123 and 79) ^b^	233 (248 and 217) ^b^	2
α_6_β_2_δ	∼300,000 [∼3.5] ^a^	2420 ± 210 ^a^	5
α_6_β_2_	∼300,000 [∼3.5] ^a^	2350 ± 200 ^a^	4
Clonazepam			
α_1_β_2_γ_2S_	15 [7.81 ± 0.12]	209 ± 14	4
α_2_β_2_γ_2S_	7.4 [8.13 ± 0.04]	253 ± 17	5
α_3_β_2_γ_2S_	16 (20 and 13) ^b^	316 (334 and 297) ^b^	2
α_5_β_2_γ_2S_	21 [7.68 ± 0.17]	255 ± 25	5
α_6_β_2_δ	29,000 [4.53 ± 0.10]	283 ± 38	5

^a^ The concentration-response curves for *N*-desmethylclobazam at the α_6_β_2_δ and α_6_β_2_GABA_A_Rs were not saturated at the maximal concentration used (1 mM). Thus, for this receptors the EC_50_ and pEC_50_ values for *N*-desmethylclobazam are estimated from the data, and the currents evoked by 1 mM *N*-desmethylclobazam (in % of the GABA EC_20_ response) is given instead of I_max_.

^b^ The properties for *N*-desmethylclobazam at α_5_β_2_γ_2S_ and for clonazepam at α_3_β_2_γ_2S_ are based on two independent experiments (n = 2), and thus the mean EC_50_ and I_max_ values for the modulators are given with specific EC_50_ and I_max_ values determined in the two experiments in parantheses.

EC_50_ values are given in nM with pEC_50_ ± S.E.M. values in brackets, I_max_ values are given as % of the response evoked by GABA EC_20_ at the receptor, and the numbers of experiments (N) are also given.

Clobazam, *N*-desmethylclobazam, and clonazepam all potentiated GABA EC_20_-mediated responses through the α_1_β_2_γ_2S_, α_2_β_2_γ_2S_, α_3_β_2_γ_2S_ and α_5_β_2_γ_2S_ GABA_A_Rs in a reversible and concentration-dependent manner (Fig. 3A and 3B). Clobazam and *N*-desmethylclobazam displayed EC_50_ values within the 100–300 nM range at all four receptors, whereas clonazepam was 9-, 19-, 15-, and 8-fold more potent than clobazam and 10-, 19-, 18-, and 5-fold more potent than *N*-desmethylclobazam as a PAM at α_1_β_2_γ_2S_, α_2_β_2_γ_2S_, α_3_β_2_γ_2S_ and α_5_β_2_γ_2S_, respectively (Table 2). In terms of modulatory efficacy, saturating concentrations of clobazam and *N*-desmethylclobazam potentiated the GABA EC_20_-evoked currents through the receptors 203–270%, corresponding to 40–54% of the maximum responses evoked by GABA through the respective receptors. Clonazepam potentiated the GABA EC_20_-evoked responses through the receptors of 209–316%, corresponding to 42–63% of the maximum GABA responses (Fig. 3B, Table 2).

Statistical evaluation of the differences in pEC_50_ and I_max_ values exhibited by clobazam, *N*-desmethylclobazam, and clonazepam at the four α_1,2,3,5_β_2_γ_2S_ GABA_A_R subtypes was performed (Table 3). Significant differences (*P*<0.05) were identified for the pEC_50_ values for clobazam between α_1_β_2_γ_2S_ and α_3_β_2_γ_2S_ subtypes as well as between the α_2_β_2_γ_2S_ and α_3_β_2_γ_2S_ receptors. The I_max_ value of clobazam at α_5_β_2_γ_2S_ was significantly smaller than those at α_1_β_2_γ_2S_ (*P*<0.05), α_2_β_2_γ_2S_ (*P*<0.01), and α_3_β_2_γ_2S_ (*P*<0.01). The pEC_50_ value of *N*-desmethylclobazam at α_3_β_2_γ_2S_ was significantly smaller than those at α_2_β_2_γ_2S_ (*P*<0.01) and α_5_β_2_γ_2S_ (*P*<0.05), and its I_max_ value at α_1_β_2_γ_2S_ was significantly smaller than those obtained at α_2_β_2_γ_2S_ and α_3_β_2_γ_2S_ receptors (*P*<0.05). As for the functional properties of clonazepam at the four receptors, only a significant difference was identified for its I_max_ values at α_3_β_2_γ_2S_ and α_1_β_2_γ_2S_ (*P*<0.05).

**Table 3:** Statistical analysis of the functional properties of clobazam, *N*-desmethylclobazam, and clonazepam at the human α_1_β_2_γ_2S_, α_2_β_2_γ_2S_, α_3_β_2_γ_2S_ and α_5_β_2_γ_2S_ GABA_A_Rs.

pEC_50_ Values														
Clobazam	α_1_β_2_γ_2S_	α_2_β_2_γ_2S_	α_3_β_2_γ_2S_	α_5_β_2_γ_2S_	*N*-desmethylclobazam	α_1_β_2_γ_2S_	α_2_β_2_γ_2S_	α_3_β_2_γ_2S_	α_5_β_2_γ_2S_	Clonazepam	α_1_β_2_γ_2S_	α_2_β_2_γ_2S_	α_3_β_2_γ_2S_	α_5_β_2_γ_2S_
α_1_β_2_γ_2S_	—	NS	*P*<0.05	NS	α_1_β_2_γ_2S_	—	NS	NS	NS	α_1_β_2_γ_2S_	—	NS	NS	NS
α_2_β_2_γ_2S_	—	—	*P*<0.05	NS	α_2_β_2_γ_2S_	—	—	*P*<0.01	NS	α_2_β_2_γ_2S_	—	—	NS	NS
α_3_β_2_γ_2S_	—	—	—	NS	α_3_β_2_γ_2S_	—	—	—	*P*<0.05	α_3_β_2_γ_2S_	—	—	—	NS
α_5_β_2_γ_2S_	—	—	—	—	α_5_β_2_γ_2S_	—	—	—	—	α_5_β_2_γ_2S_	—	—	—	—
I_max_ Values														
Clobazam	α_1_β_2_γ_2S_	α_2_β_2_γ_2S_	α_3_β_2_γ_2S_	α_5_β_2_γ_2S_	*N*-desmethylclobazam	α_1_β_2_γ_2S_	α_2_β_2_γ_2S_	α_3_β_2_γ_2S_	α_5_β_2_γ_2S_	Clonazepam	α_1_β_2_γ_2S_	α_2_β_2_γ_2S_	α_3_β_2_γ_2S_	α_5_β_2_γ_2S_
α_1_β_2_γ_2S_	—	NS	NS	*P*<0.05	α_1_β_2_γ_2S_	—	*P*<0.05	*P*<0.05	NS	α_1_β_2_γ_2S_	—	NS	*P*<0.05	NS
α_2_β_2_γ_2S_	—	—	NS	*P*<0.01	α_2_β_2_γ_2S_	—	—	NS	NS	α_2_β_2_γ_2S_	—	—	NS	NS
α_3_β_2_γ_2S_	—	—	—	*P*<0.01	α_3_β_2_γ_2S_	—	—	—	NS	α_3_β_2_γ_2S_	—	—	—	NS
α_5_β_2_γ_2S_	—	—	—	—	α_5_β_2_γ_2S_	—	—	—	—	α_5_β_2_γ_2S_	—	—	—	—

*P*-values from one-way ANOVA testing if mean values (pEC_50_ and I_max_) experimentally determined for the compounds with Tukey’s Multiple Comparison *Post-hoc* Test. NS; not significant.

### Investigations into the benzodiazepine-mediated modulation of the α_1,2,3,5_β_2_γ_2S_ GABA_A_Rs

**Fig 4:**
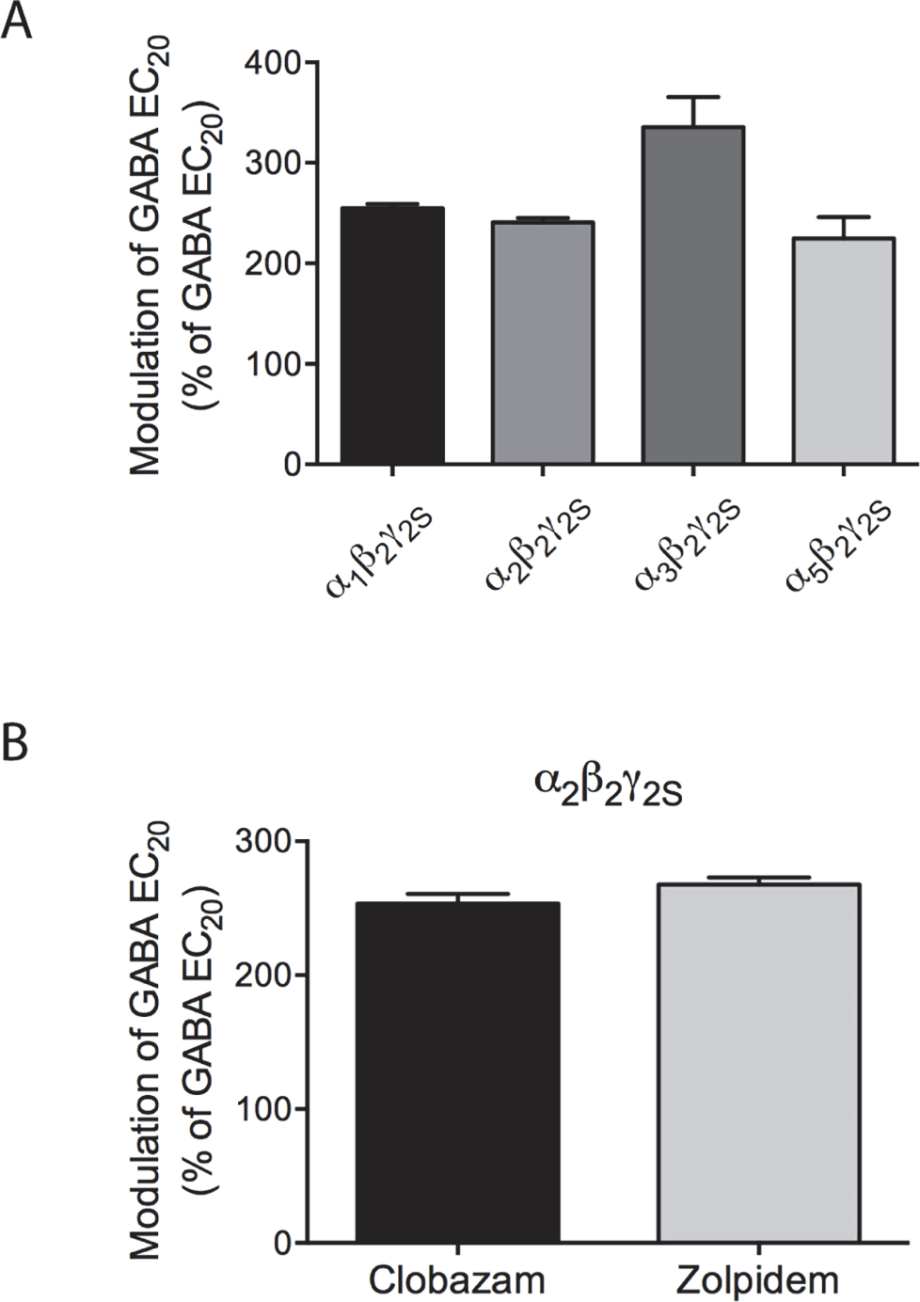
Comparison of the functional efficacies of clobazam, *N*-desmethylclobazam, and clonazepam at α_1,2,3,5_β_2_γ_2S_ GABA_A_Rs with those of diazepam and zolpidem. (A) Potentiation of the response elicited by GABA EC_20_ by 3 μM diazepam in Xenopus oocytes injected with cRNAs encoding for α_1_β_2_γ_2S_, α_2_β_2_γ_2S_, α_3_β_2_γ_2S_ and α_5_β_2_γ_2S_ GABA_A_Rs in a subunit ratio of 1:1:1 (means ± S.E.M.; N = 2–4) (B) Potentiation of the response elicited by EC_20_ GABA by 3 μM clobazam and 3 μM zolpidem in Xenopus oocytes injected with cRNAs encoding the α_2_β_2_γ_2S_ GABA_A_R injected in a subunit ratio of 1:1:5 (means ± S.E.M.; N = 2).

As will be outlined in the *Discussion* section, the modulatory efficacies exhibited by benzodiazepines at αβγ GABA_A_Rs in recombinant expression systems in previous studies have varied considerably. To address this aspect, we compared the maximum responses mediated by saturating concentrations (3 μM) of clobazam, *N*-desmethylclobazam, and clonazepam at the GABA_A_Rs with the maximal responses mediated by a saturating concentration of diazepam, a 1,4-benzodiazepine often referred to as a “full benzodiazepine agonist”. The maximum responses evoked by clobazam and *N*-desmethylclobazam when co-applied with GABA EC_20_ at α_1_β_2_γ_2S_ and α_2_β_2_γ_2S_ GABA_A_Rs, respectively, were slightly but significantly smaller than those mediated by diazepam at the respective receptors (p<0.1; ordinary one-way ANOVA) (Table 2, Figs. 3B and 4A). In contrast, the maximum responses mediated by clobazam at α_2,3,5_β_2_γ_2S_, by *N*-desmethylclobazam at α_1,3,5_β_2_γ_2S_ and by clonazepam at all the four receptors did not differ significantly from those exhibited by diazepam (Table 2, Figs. 3B and 4A). We also compared the maximum degree of potentiation mediated by clobazam at the α_2_β_2_γ_2S_ GABA_A_R with the maximal responses induced by a saturating concentration of zolpidem. Albeit not a benzodiazepine, zolpidem acts as a PAM through the high-affinity benzodiazepine site in α_1,2,3,5_βγ GABA_A_Rs, and the compound has previously been reported to be a “full benzodiazepine-site agonist” at the α_2_β_2_γ_2_ subtype [52]. In this experiment cRNA for α_2_β_2_γ_2S_ was injected in a ratio of 1:1:5 (α_2_:β_2_:γ_2S_) to facilitate the expression of a homogenous population of γ_2S_-containing complexes. The modulatory efficacies displayed by clobazam and zolpidem at the α_2_β_2_γ_2S_ receptors in these experiments did not differ significantly (Fig. 4B). Moreover, the respective degrees of potentiation mediated by clobazam and zolpidem at the receptors expressed in these oocytes did not differ significantly from those at oocytes injected with an α_2_:β_2_:γ_2S_ cRNA ratio of 1:1:1 (Fig. 4B and data not shown).

**Fig 5:**
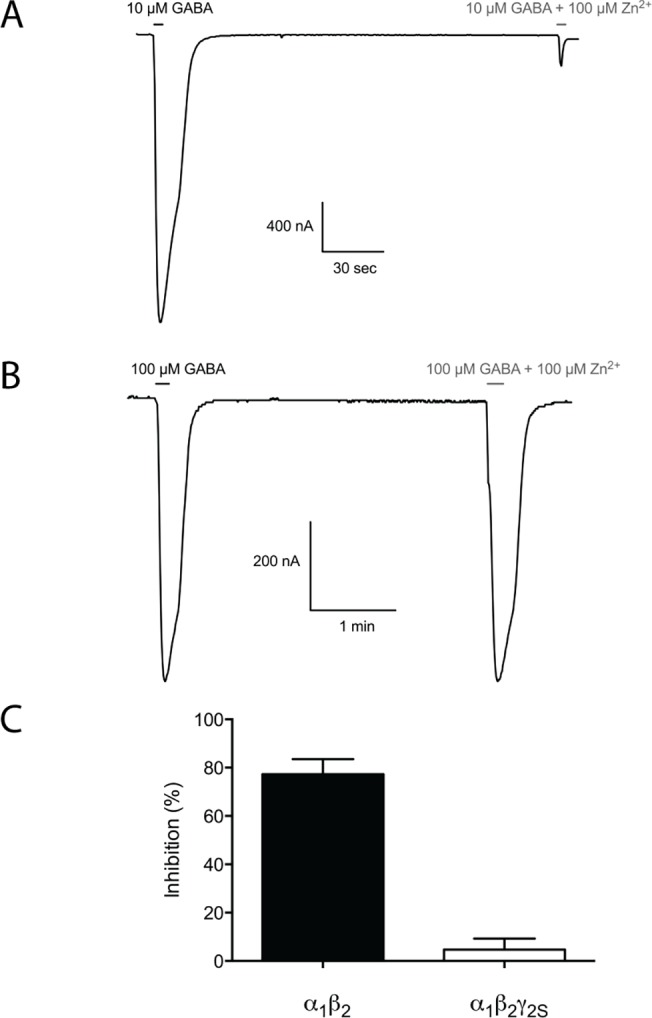
Zinc-mediated inhibition of human α_1_β_2_ and α_1_β_2_γ_2S_ GABA_A_R signalling in *Xenopus* oocytes. (A) Representative trace of the inhibition mediated 100 μM Zn^2+^ of the currents elicited by 10 μM GABA (EC_80_) through the α_1_β_2_ GABA_A_R. (B) Representative trace of the inhibition mediated 100 μM Zn^2+^ of the currents induced by 100 μM GABA (EC_80_) through the α_1_β_2_γ_2S_ GABA_A_R. (C) The degree of inhibition mediated by 100 μM Zn^2+^ of GABA EC_80_-evoked currents in oocytes expressing α_1_β_2_ mean ± S.E.M.; 77 ± 6.3%; N = 7and α_1_β_2_γ_2S_ (mean ± S.E.M.; 4.7 ± 4.6%; N = 6) GABA_A_Rs.

In another series of experiments, we took advantage of the well-documented ability of zinc to discriminate between αβand αβγ GABA_A_Rs [46, 53, 54] to investigate whether oocytes injected with α_1,2,3,5_β_2_γ_2S_ cRNAs in a 1:1:1 subunit ratio express homogeneous populations of ternary receptor complexes. As can be seen from Fig. 5, the GABA EC_80_-evoked response through the α_1_β_2_ GABA_A_R was almost completely eliminated by 100 μM Zn^2+^, whereas the presence of this concentration of the metal ion had negligible effect on the currents elicited in α_1_β_2_γ_2S_-expressing oocytes. This strongly suggests that the functional properties of clobazam, *N*-desmethylclobazam and clonazepam at the α_1,2,3,5_β_2_γ_2S_ GABA_A_Rs have been determined at homogenous populations of γ_2S_-containing receptors.

### Functional properties of clobazam, *N*-desmethylclobazam, and clonazepam at the human α_6_β_2_δ GABA_A_R expressed in *Xenopus* oocytes

**Fig 6:**
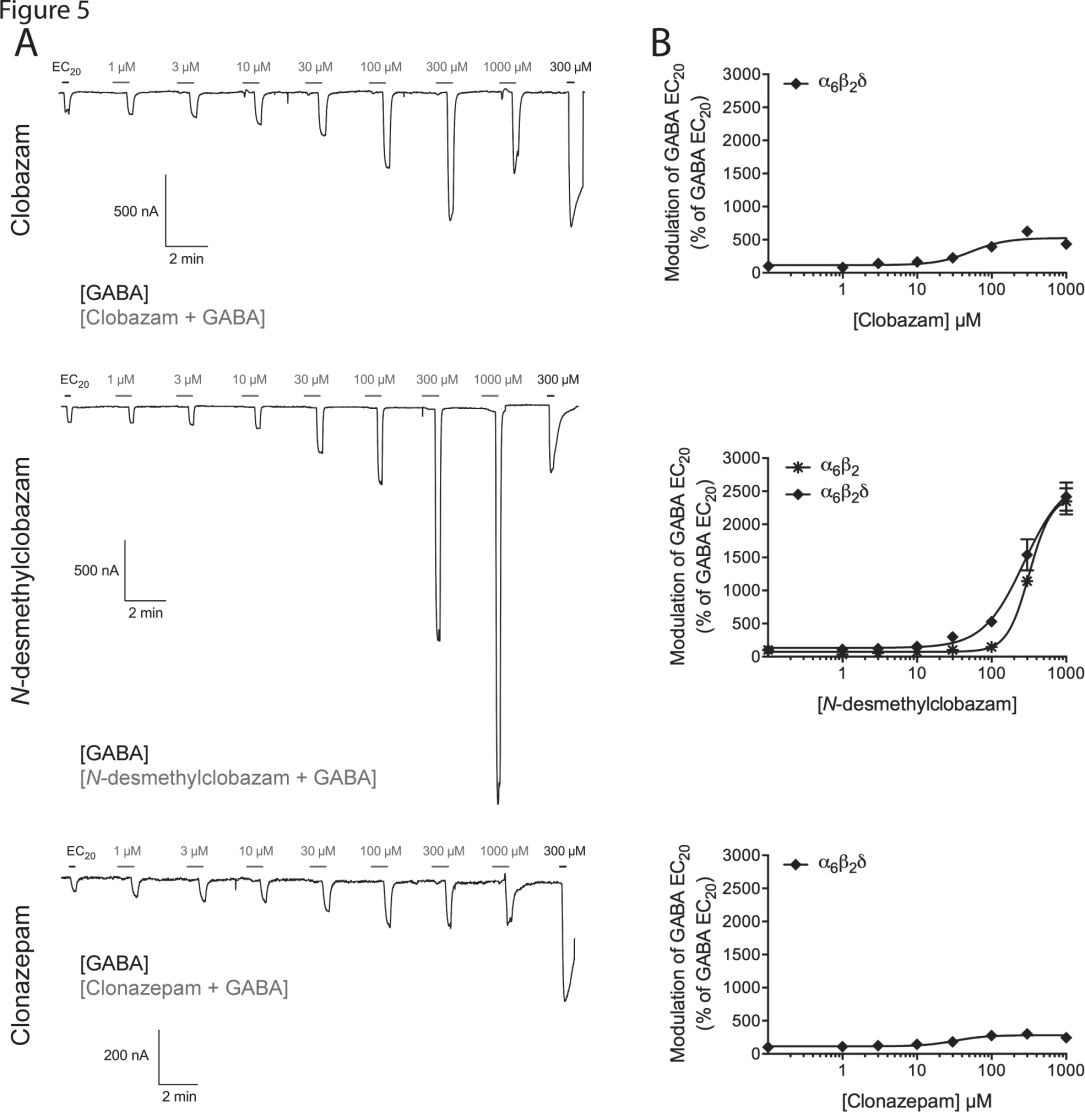
. Functional properties of clobazam, *N*-desmethylclobazam and clonazepam at the human α_6_β_2_δ and α_6_β_2_ GABA_A_Rs expressed in *Xenopus* oocytes. (A) Representative traces for various concentrations of clobazam (top), *N*-desmethylclobazam (middle) and clonazepam (bottom) co-applied with GABA EC_20_ to oocytes expressing the α_6_β_2_δ GABA_A_R. The black bars represent applications of GABA EC_20_ and of 300 M GABA that elicits maximal current through the receptor. The grey bars represent applications of various concentrations of clobazam, *N*-desmethylclobazam or clonazepam (a 30 s pre-incubation with the compound followed by co-application of the compound and GABA EC_20_). (B) Concentration-response relationships for clobazam (top), *N*-desmethylclobazam (middle) and clonazepam (bottom) at the α_6_β_2_δ GABA_A_R and for *N*-desmethylclobazam at the α_6_β_2_ GABA_A_R (middle) in the presence of GABA EC_20_ (means ± S.E.M.; N = 4–6).

To investigate whether the functional properties of clobazam and its metabolite at αβδ GABA_A_Rs potentially differ from those of clonazepam, the three compounds were tested at a representative of these receptors, the human α_6_β_2_δ subtype (Table 2). Representative traces recorded for clobazam, *N*-desmethylclobazam and clonazepam when pre-incubated and co-applied with GABA EC_20_ at the receptor are presented in Fig. 6A, and concentration-response relationships determined for the compounds at the receptor are given in Fig. 6B.

All three modulators potentiated GABA EC_20_-mediated signaling in α_6_β_2_δ-oocytes in a concentration-dependent and reversible manner, exhibiting EC_50_ values in the mid-to-high micromolar range at the receptor (Table 2). Thus, the modulatory potencies of clobazam, *N*-desmethylclobazam and clonazepam at this receptor were 200–400, 1100–3100 and 1400–3900 fold lower than those exhibited by the respective benzodiazepines at the α_1,2,3,5_β_2_γ_2S_ GABA_A_Rs, respectively (Table 2). Strikingly, however, the modulatory efficacies of the two 1,5-benzodiazepines at this receptor were very different from those at the four α_1,2,3,5_β_2_γ_2S_ receptors. Whereas the degree of potentiation of the GABA EC_20_-evoked response through α_6_β_2_δ mediated by clonazepam was comparable to those at the α_1,2,3,5_β_2_γ_2S_ GABA_A_Rs, clobazam and in particular *N*-desmethylclobazam were much more efficacious PAMs at the receptor (Table 2, Fig. 6). Albeit the concentration-response relationship for *N*-desmethylclobazam at the α_6_β_2_δ receptor was not saturated within the concentration range tested, 1 mM of the drug was found to potentiate the GABA EC_20_-evoked response through the receptor by ∼24-fold (Fig. 6A and 6B). Interestingly, a distinct inhibition phase in the concentration-response relationship was observed in some of these recordings for clobazam and clonazepam, with 1 mM of the modulator resulting in a lower degree of potentiation than 300 μM (Fig. 6A). Moreover, rebound currents were observed at these high modulator concentrations (Fig. 6A).

To elucidate the molecular basis for the high-efficacious modulation exerted by *N*-desmethylclobazam at the α_6_β_2_δ GABA_A_R, we characterized the functional characteristics of the compound at the binary α_6_β_2_ receptor. *N*-desmethylclobazam also potentiated the GABA EC_20_-mediated signaling through this receptor in a concentration-dependent manner, exhibiting modulatory potency and efficacy not significantly different from those displayed at α_6_β_2_δ (Table 2, Fig. 6B). However, it should be stressed that the EC_50_ and I_max_ values given for *N*-desmethylclobazam at α_6_β_2_δ and α_6_β_2_ are estimates, since neither of the concentration-response curves for the modulator at these two receptors reached saturation.

## Discussion

In view of the clinical use of clobazam for the treatment of various diseases over the last decades, surprising little is known about the *in vitro* pharmacology of the drug. In the present study, we have performed an elaborate functional characterization of clobazam, its major active metabolite *N*-desmethylclobazam and the 1,4-benzodiazepine clonazepam at the human α_1_β_2_γ_2S_, α_2_β_2_γ_2S_, α_3_β_2_γ_2S_, α_5_β_2_γ_2S_ and α_6_β_2_δ GABA_A_Rs expressed in *Xenopus* oocytes. A detailed functional characterization of clonazepam at recombinant GABA_A_Rs has to our knowledge not been published previously, and thus this study provides substantial insights into the molecular pharmacology of all three modulators.

Each of the three benzodiazepines exhibited similar EC_50_ values as a PAM at the four α_1,2,3,5_β_2_γ_2S_ GABA_A_Rs, and the maximum responses evoked by saturating concentrations of each of the modulators upon co-application with GABA EC_20_ at these four subtypes were also very similar (Table 2). Although statistical analysis identified differences between some of the potencies and efficacies displayed by the respective compounds at the four receptors (Table 3), these differences are not considered pertinent from a biological perspective. It is important to stress that the α_1,2,3,5_β_2_γ_2S_ receptors included in this study only constitute a selection of all αβγ receptors targeted by benzodiazepines in clinically relevant concentrations. However, the fact that benzodiazepines have been reported to be less potent and less efficacious modulators of αβγ_1_ and αβγ_3_ receptors than of the corresponding αβγ_2_ receptors [55–57] combined with the very restricted expression of γ_1_ and γ_3_ in the CNS [58, 59] strongly suggests that the contributions of these receptor assemblies to the overall clinical effects of benzodiazepines are negligible. More importantly, the identity of the β subunit in the αβγ_2_ GABA_A_R complex is not believed to influence benzodiazepine pharmacology substantially [56, 57], just as there to our knowledge are no reports of benzodiazepines exhibiting significantly different pharmacological properties at γ_2S_- and γ_2L_-containing GABA_A_Rs. Thus, although we can not exclude the possibility that the functional properties of clobazam, *N*-desmethylclobazam and/or clonazepam at α_1,2,3,5_βγ_2_ complexes comprising β_1_β_3_ and/or γ_2L_ subunits could differ from those observed at the receptors in this study, we propose that the three benzodiazepines are likely to act as non-selective PAMs at all α_1,2,3,5_βγ_2_ receptors.

In our recent study of the binding properties of the three benzodiazepines, clobazam and *N*-desmethylclobazam displayed slightly but significantly higher binding affinities at the α_2_β_2_γ_2S_ GABA_A_R compared to the α_1_β_2_γ_2S_ subtype (2.5- and 4.3-fold, respectively), whereas clonazepam exhibited significantly higher binding affinities to α_1,2,5_β_2_γ_2S_ subtypes than to the α_3_β_2_γ_2S_ receptor (2.8- to 3.4-fold) [41]. The fact that these binding subtype-preferences are not mirrored in the functional profiles of the modulators is not particular surprising, given the different methodologies used to assess binding affinities and functional potencies of modulators. In support of this, another interesting observation that can be extracted from the two studies is that the 200–1000 fold higher binding affinities displayed by clonazepam compared to clobazam and *N*-desmethylclobazam at the four α_1,2,3,5_β_2_γ_2S_ receptors in the [^3^H]flumazenil binding assay translate into considerably less pronounced differences (5–19 fold) between the EC_50_ values of the 1,4-benzodiazepine and the two 1,5-benzodiazepines at the receptors in the oocyte recordings (Table 2) [41]. These observations may reflect differences in the degrees of receptor desensitization in the two assays.

A comprehensive literature search has only identified two previous studies of the functional properties of clobazam and *N*-desmethylclobazam at recombinant GABA_A_Rs [55, 60]. Clobazam has been reported to exert negligible potentiation of GABA EC_5_-EC_10_-evoked responses through α_1_β_2_γ_1_ receptors in *Xenopus* oocytes, suggesting that the presence of this γ subunit in the GABA_A_R complex alters the functionality of the 1,5-benzodiazepine substantially [55]. Of particular interest for this study, Fisher has performed a direct comparison of the modulation mediated by clonazepam, clobazam, and *N*-desmethylclobazam on the current elicited by 3 μM GABA (EC_10_–EC_20_) through the rat α_3_β_3_γ_2L_ GABA_A_R expressed in HEK-293T cells by patch clamp electrophysiology [60]. The EC_50_ values determined for clonazepam, clobazam, and *N*-desmethylclobazam at the rat α_3_β_3_γ_2L_ receptor in the Fisher study were 90 nM, 490 nM and 550 nM, respectively, which are in good agreement with the potencies exhibited by three benzodiazepines at the human α_3_β_2_γ_2S_ receptor in this study (Table 2) [60]. However, Fisher found clobazam and diazepam to be substantially more efficacious potentiators of rat α_3_β_3_γ_2L_ currents (I_max_ values of 487% and 508% of the responses evoked by GABA EC_10_–EC_20_, respectively) than *N*-desmethylclobazam and clonazepam (I_max_ values of 270% and 263%, respectively) [60]. Whereas the modulatory efficacies of *N*-desmethylclobazam and clonazepam at α_3_β_3_γ_2L_ are in good agreement with those at the α_3_β_2_γ_2S_ receptor in the present study, the higher efficacies exhibited by clobazam and diazepam in the Fisher study clearly contrast the comparable I_max_ values determined for the four PAMs at the α_3_β_2_γ_2S_ receptor in this study (Table 2, Figs. 3 and 4). Several factors might explain this apparent discrepancy, including the different receptors studied (rat α_3_β_3_γ_2L_ vs. human α_3_β_2_γ_2S_), the different expression systems (HEK-293T cells vs. *Xenopus* oocytes), and the different recording techniques (patch clamp vs. TEVC electrophysiology). We propose that the precise determination of the GABA EC_20_ used for the characterization of the benzodiazepines in the present study can have facilitated a more precise determination of the degree of maximum potentiation of the GABA-evoked responses than the GABA EC_10_–EC_20_ concentrations employed in the Fisher study. On the other hand, the faster application rate of the compounds in patch clamp recordings using HEK-293T cells may have resulted in less concomitant desensitization of the receptors during application than in the *Xenopus* oocyte recordings system, which is characterized by slower exchange rates. Thus, we cannot exclude the possibility that concurrent desensitization of the receptors upon co-application of GABA EC_20_ with high benzodiazepine concentrations could constitute a ceiling effect with respect to the degree of maximum response elicited by the benzodiazepine-bound receptor, and that this effect potentially can have masked putative differential efficacies of the benzodiazepines at the receptors. However, as will be outlined below, the divergent efficacies reported for benzodiazepines at GABA_A_Rs expressed in oocytes in the literature strongly suggest that establishing a “true efficacy” for any given benzodiazepine is not trivial.

In the absence of other previous studies of the functional characteristics of clobazam, *N*-desmethylclobazam and/or clonazepam at GABA_A_Rs expressed in *Xenopus* oocytes, we investigated whether the determined modulatory efficacies for these compounds could be considered reliable by comparing them to the efficacies mediated by two reference benzodiazepine-site modulators. A saturating concentration of the prototypic benzodiazepine diazepam was observed to induce comparable or slightly higher responses than those mediated by saturating concentrations of clobazam, *N*-desmethylclobazam and clonazepam at the four α_1,2,3,5_β_2_γ_2S_ subtypes (Figs. 3B and 4A). Furthermore, the maximum responses induced by zolpidem at α_1_β_2_γ_2S_ and α_2_β_2_γ_2S_ receptors were comparable to those mediated by clobazam (Fig. 4B and data not shown). Unfortunately, the efficacies determined for diazepam and zolpidem at GABA_A_Rs expressed in oocytes in previous studies have varied considerably. Although often referred to as a “full benzodiazepine agonist,” diazepam has exhibited very different efficacies as a PAM of GABA EC_10_- to EC_20_-evoked currents through α_1_β_2_γ_2_ receptors in previous studies, including degrees of potentiation in the same 2.3- to 3.4-fold range as exhibited by the modulator at the α_1,2,3,5_β_2_γ_2S_ receptors in this study [61–63]. Likewise, whereas zolpidem has been reported to potentiate the GABA EC_15_- to EC_20_-evoked responses through α_1_β_2_γ_2_ and α_2_β_2_γ_2_ receptors 4- to 6-fold in some studies [52, 64, 65], its maximum modulation of GABA-evoked currents through the receptors in other studies have been very similar to the 2.7-fold potentiation observed at α_2_β_2_γ_2S_ in this study [61, 62, 66]. In conclusion, the results in this study indicate that clobazam and *N*-desmethylclobazam are equally efficacious or almost as efficacious as the 1,4-benzodiazepines clonazepam and diazepam at the human α_1,2,3,5_β_2_γ_2S_ receptors. However, considering the variation in the efficacies reported for standard benzodiazepines in the literature, the absolute degrees of potentiation exerted by the two 1,5-benzodiazepines at the receptors in other recording set-ups could potentially differ from those observed in this study.

The α_6_β_2_δ receptor was included in this study as a representative of the δ-containing GABA_A_Rs, and clobazam, *N*-desmethylclobazam and clonazepam all displayed mid-to-high micromolar potencies as PAMs of this receptor (Fig. 6, Table 2). Apart from the obvious absence of an α^(+)^/γ^(–)^ subunit interface in the α_6_β_2_δ complex, the distinct functional characteristics exhibited by the three benzodiazepines at this receptor support the notion of them acting through another allosteric site than the classical high-affinity benzodiazepine binding site, and the comparable potencies displayed by *N*-desmethylclobazam as a PAM at the α_6_β_2_ and α_6_β_2_δ receptors strongly suggest that this site is comprised within the αβ regions of the αβδ complex (Fig. 6B, Table 2). Some of the characteristics displayed by the benzodiazepines at the α_6_β_2_δ GABA_A_R could be argued to be indicative of a binding site in the transmembrane domain of the receptor. The tendencies towards bell-shaped concentration-response curves observed for clobazam and clonazepam as well as the rebound currents observed at high concentrations of the modulators are certainly reminiscent of the characteristics previously reported for PAMs/ago-PAMs acting through transmembrane domains of GABA_A_Rs [55, 67–72]. Moreover, whereas PAMs targeting extracellular non-cannonical subunit interfaces in GABA_A_Rs and other Cys-loop receptors historically predominantly have been found to increase agonist potency without affecting the maximal agonist response through the receptors significantly [20, 73, 74], several PAMs acting through the transmembrane domains of the receptors have been shown capable of increasing agonist-evoked maximal peak currents, analogously to the high-efficacious potentiation of α_6_β_2_δ signaling mediated by *N*-desmethylclobazam [67, 74–76]. However, this phenotypic difference between PAMs acting through extracellular and transmembrane regions of Cys-loop receptors does not appear to be black-and-white, as illustrated by the pronounced enhancement of GABA efficacy at the α_1_β_3_δ GABA_A_R mediated by LAU 177 through a site in the extracellular α_1_^(+)^/β_3_^(–)^ interface [22] and by the augmentation of agonist efficacy exerted by the anthelmintic drug morantel through the extracellular β_2_^(+)^/α_3_^(–)^ interface of the α_3_β_2_ nicotinic acetylcholine receptor [77]. Thus, not having explored the modes of action of clobazam, *N*-desmethylclobazam and clonazepam at the α_6_β_2_δ GABA_A_R in detail, we will refrain from speculations about whether the modulators target a low-affinity binding site located in the transmembrane domain, in the extracellular α^(+)^/β^(–)^ subunit interface, or in another region of the receptor complex.

The nature and efficacies of the modulation exerted by clobazam and *N*-desmethylclobazam at other αβ and αβδ receptors could potentially differ from those observed for the compounds at α_6_β_2_ and α_6_β_2_δ. However, the two 1,5-benzodiazepines are likely to be weak modulators at all αβand αβδ GABA_A_Rs, thus mediating their effects at these receptors at substantially higher concentrations than those required to modulate αβγ receptors. Although the μM activity component of benzodiazepines previously has been proposed to contribute to the CNS depression observed at high *in vivo* concentrations [18], we find it highly improbable that this component contributes significantly to the clinical efficacy of clobazam/*N*-desmethylclobazam. On the other hand, the highly efficacious potentiation of α_6_β_2_δ GABA_A_R signaling exerted by *N*-desmethylclobazam is quite interesting from a molecular perspective, and we propose that the 1,5-benzodiazepine could constitute an interesting lead scaffold for the development of novel allosteric modulators targeting this elusive low-affinity benzodiazepine binding site in the GABA_A_Rs.

## Conclusion

The present study represents the first elaborate *in vitro* functional characterization of clobazam, *N*-desmethylclobazam and clonazepam at recombinant human GABA_A_Rs. While both 1,5-benzodiazepines are potent PAMs of human α_1,2,3,5_β_2_γ_2S_ receptors, they appear to be non-selective both in terms of potency and efficacy, a characteristic they share with many classical 1,4-benzodiazepines including clonazepam (Table 2) [15, 16]. Obviously we can not completely exclude the possibility that the functionalities of clobazam and/or *N*-desmethylclobazam at one or a few of the GABA_A_R subtypes not included in this study could differ substantially from those of clonazepam and other 1,4-benzodiazepines, and that this difference could contribute to the distinct properties observed for clobazam compared with the classical 1,4-benzodiazepines in the clinic. However, judging from the results in this study we propose that these clinical differences are more likely to be rooted in other factors than the *in vitro* pharmacological properties of the modulators, such as their respective pharmacokinetic characteristics.
